# Prioritization of hazards for risk and resilience management through elicitation of expert judgement

**DOI:** 10.1007/s11069-022-05287-x

**Published:** 2022-04-20

**Authors:** Ioanna Ioannou, Jaime E. Cadena, Willy Aspinall, David Lange, Daniel Honfi, Tiziana Rossetto

**Affiliations:** 1 EPICentre, Department of Civil, Environmental and Geomatic Engineering, University College London, UCL, London, UK; 2 School of Civil Engineering, University of Queensland, Brisbane, Australia; 3 School of Earth Sciences, University of Bristol, Bristol, UK; 4 Monitoring and Analyses of Existing Structures, Ramboll, Copenhagen, Denmark

**Keywords:** Paired comparison, Stakeholders, Natural hazards, Operational hazards

## Abstract

Risk assessment in communities or regions typically relies on the determination of hazard scenarios and an evaluation of their impact on local systems and structures. One of the challenges of risk assessment for infrastructure operators is how to identify the most critical scenarios that are likely to represent unacceptable risks to such assets in a given time frame. This study develops a novel approach for prioritizing hazards for the risk assessment of infrastructure. Central to the proposed methodology is an expert elicitation technique termed paired comparison which is based on a formal mathematical technique for quantifying the range and variance in the judgements of a group of stakeholders. The methodology is applied here to identify and rank natural and operational hazard scenarios that could cause serious disruption or have disastrous effects to the infrastructure in the transnational Øresund region over a period of 5 years. The application highlighted substantial divergences of views among the stakeholders on identifying a single ‘most critical’ natural or operational hazard scenario. Despite these differences, it was possible to flag up certain cases as critical among the natural hazard scenarios, and others among the operational hazards.

## Introduction

Infrastructure assets and systems may be exposed to a plethora of hazard scenarios, such as natural hazard events (e.g., earthquakes or floods), operational accident (e.g., malfunction of equipment) or market/economy hazard (e.g., bankruptcy of main user of the facility, war, etc.), which can affect an infrastructure system or interconnected systems. Ideally, the vulnerability of infrastructure assets and the resulting impact should be evaluated against a wide range of plausible scenarios. However, in practice, time constraints, limited resources, and multiple unknown (Park et al. 2013) or partially understood hazards mean that this task may not be feasible and that prioritization in planning or mitigation may be necessary in response to only one or two hazards.

Risk management standards such as ISO 31000 highlight the importance of identifying critical hazard scenarios to be able to inform the rest of the risk assessment process (Standardization 2018). Given that the validity of the risk assessment outputs depends on these scenarios, techniques such as hazards and operability analysis, failure mode and effect analysis and the logical constructions provided by the fault and event tree analysis are often used (Kaplan et al. 2001). These are known as hazard identification techniques (Center for Chemical Process 2010) and are suitable for different systems at different stages of operation. However, these are not the only approaches to construct scenarios and there are many which recognize the high complexity of systems, the role of stakeholders and the dependency between different systems and variables, which are often ignored (Underwood and Waterson 2014).

However, when evaluating the possible impact of failures in infrastructure systems, the wider consequences must be considered. The World Economic Forum global risks report highlights the potential for systemic dependencies to result in cascading failures between interconnected infrastructures increasing the potential consequences of damage to one system (McLennan 2021). These often-unpredictable cascading failures between infrastructure systems therefore have the potential to cause significant impact on affected communities. Such interdependencies are one of two issues which were identified in the 2012 review of the European Program for Critical Infrastructure Protection, the other is increasing the resilience of Critical Infrastructure (Commission 2012).

Numerous frameworks for resilience management with potential application to infrastructure have been developed, for example (Tierney and Bruneau 2007; Miles and Chang 2011; Petit et al. 2013; Lange et al. 2017; Hernantes et al. 2019; Rød et al. 2020). This latter framework formalizes definitions associated with the resilience management process by mapping the equivalent definitions from the risk management process and is thus compatible with the risk management approach of ISO 31000 (Standardization 2018). However, regardless of the approach taken to risk or resilience management, where such an analysis has as an antecedent the vulnerability of infrastructure to a specific event, then a key step in the overall process is the identification of hazard scenarios that might disrupt the function of the infrastructure, affect its integrity and have broader and, perhaps long-lasting, local, national or international consequences.

Consequently, as a result of the need to account for interdependencies and cascading effects between different systems, for infrastructure investment and planning on a strategic level, then considering the broad of range of hazards which could affect all of the infrastructure in a specific region is of significant value for both risk and resilience management. This requires a broad understanding of not only hazards which could affect a region, but also the vulnerability of individual infrastructure sectors to these hazards. This study aims to develop a methodology which meaningfully integrates the expertize of a broad range of stakeholders including, e.g., infrastructure operators and civil protection agencies in ranking hazard scenarios to determine priority hazards to account for in risk and resilience treatment in a specific region and for interdependent infrastructures.

According to the methodology proposed, a list of pre-defined plausible scenarios is identified and these are ranked for a region through the prioritization of their perceived likelihood to cause either a disaster or an emergency from the perspective of a range of stakeholders either of an infrastructure system or in a region. This approach incorporates the specific expert knowledge of all stakeholders, which can include expert operators of a unique system or of a well-known system in a unique context. The underlying methodology is based on an expert elicitation scheme. While the expert elicitation scheme itself is not novel, its application in this context is and it allows the characterization of the risk perception of the stakeholders. Mapping stakeholders’ risk perception is an input for the risk management process of infrastructure systems (Santoro et al. 2019), and it can enhance the risk communication process (Micic 2016).

Multiple definitions of risk exist (Aven and Renn 2009; Analysis 2015), which are suitable for different contexts. The approach used in this paper defines the risk of a particular scenario based on two dimensions: its likelihood of occurrence and the severity of its impact. This is aligned with Kaplan’s definition of risk (Kaplan and Garrick 1981). Within the context of resilience of infrastructure, the consequence analysis is limited to the impact on either the infrastructures integrity or its function. Both the likelihood of occurrence and consequence analyses are challenging calculations. As presented by Goertland et al. (2016), these calculations within typical quantitative risk assessments can yield an uncertainty margin of ± 3 orders of magnitude; such a margin could render most assessments unreliable and not useful. Given these difficulties, the literature provides as an alternative the use of semi-qualitative approaches (Haimes et al. 2002; Mansouri et al. 2010; Johnsen and Veen 2013; Chang et al. 2014), where the risk scenarios are represented in a risk matrix and some form of expert judgement is used to rank them. These matrices express the risk due to a given hazard scenario in terms of a single point estimate value, obtained through an enforced or inferred consensus among the participants. Unfortunately, most such approaches ignore the degree of any divergence in judgements within the group of participants, thus discarding valuable information about uncertainties in the estimates.

The novelty of the methodology developed here therefore lies in its use of Structured Expert Judgment (SEJ) elicitation; a formalized process to determine a rational consensus among subject-matter experts on the uncertainties associated with problems where sufficient empirical or historical data are not available to characterize uncertainties statistically. It draws on the expertize of stakeholders and thus is particularly suited to complex systems, which are difficult to model, and for assessing the relative risks of unusual or exceptional events for which empirical observations are scarce this information can be as an input for risk and resilience assessment processes as well as to support the construction of risk and resilience management strategies. This has the added advantage of characterizing stakeholders risk perception and deviating from similar approaches (Ergenç and Barış 2018) it focuses on quantifying the degree of disagreement among the stakeholders.

In the following sections, the proposed methodology is described. The methodology is then demonstrated through the identification of a range of scenarios most likely to cause various levels of damage and disruption within the Øresund region over a period of 5 years as an example. This region is selected as a case study because of the unique features of this region. It represents a transnational metropolitan area around the Øresund strait between Denmark and Southern Sweden and thus integrates various operators of infrastructure and social contexts. Thus managing risks in this region may require the collaboration of a range of stakeholders and actors as well as information about how these risks are perceived by these various organizations.

## Methodology for scenario prioritization

The proposed methodology in this study aims to identify critical scenarios which can be used in a subsequent risk/resilience assessment process. A key aspect of the proposed methodology is the semi-qualitative risk assessment of each scenario, which is based on the opinions of multiple stakeholders. As stated, in the context of this study, the risk of a given scenario is defined as a function of the likelihood of an event and its consequences (Eq. 1).

1 $${\text{Risk}} = \, f\left( {{\text{Event}}\,{\text{Likelihood}},\,{\text{Consequences}}} \right)$$

where Event Likelihood represents the perceived likelihood of occurrence of a hazard scenario (i.e., for the needs of this study determined as a trigger event), which can disrupt one or more infrastructure assets. Consequences expresses the level of disruption to the service provided by the infrastructure and the impact of that service loss on society or the economy. Economic losses include both direct (e.g., cost of any physical damage caused) and indirect losses (e.g., due to loss of revenue, cost of long-term recovery, social impact, etc.).

Both dimensions of risk (i.e., Event Likelihood and Consequences) can be understood as stochastic variables often with very little support for their frequencies as a result of a paucity of statistical data for the systems and scenarios being studied. A simpler approach is taken in the literature where values to the two dimensions of risk are provided subjectively by assigning a value based on a scale, e.g. (U.S. Department of Defense 2003; NASA 2007; Cox 2008; Mansouri et al. 2010; European Commission—Joint Research Centre 2017), to the relative likelihood of occurrence of each scenario as well as to the severity of their consequences. Some studies (e.g., Mansouri et al. 2010) multiply the two values and their product expresses the risk factor that is used to prioritize the scenarios. However, it has been argued that this practice leads to meaningless results given that the values assigned lack cardinal meaning (U.S. Department of Defense 2003; Cox 2008). Another popular approach is the representation of the two dimensions in the form of a risk matrix (U.S. Department of Defense 2003; NASA 2007; European Commission - Joint Research Centre 2017). Both of these approaches incorporate a high degree of subjectivity and bias resulting from the unique experiences of the risk analyst. The limitations of such approaches to risk prioritization have been discussed in detail elsewhere, e.g. (Cox 2008), where issues relating to the poor resolution and errors in the overestimation of the risk of scenarios with low-frequency-high-consequences have been noted. Duijm (2015) provides a detailed review of the challenges associated to risk matrices and also recommendations for their design and use.

In the proposed methodology in this paper, the perceived likelihood of occurrence of a scenario, as well as the likelihood of this scenario to cause a given level of consequence, is ranked through a rational consensus of multiple experts reached by the use of paired comparison based on a formal mathematical technique termed probabilistic inversion (Cooke and Misiewicz 2007). Problems that may benefit from probabilistic inversion can arise when quantifying uncertainty in physical models with expert judgement (Kraan and Bedford 2005), i.e., the type of problems discussed in the previous paragraph. The goal of probabilistic inversion is to quantify the uncertainty on parameters of some model using expert judgement, usually when the model parameters do not possess a clear physical meaning; hence, the experts are confronted with measurements or observations that do not possess familiar physical units. Moreover, the model of interest may be derived under assumptions with which the experts do not necessarily agree. The challenge then is to elicit expert assessments of defined observable quantities, i.e., “output” values that are functionally related with the model parameters in other words, it is an inverse problem involving a number of expert evaluations, each of which is credible to the individual expert but will likely differ between experts.

The process may be characterized as pushing the stakeholders’ output judgements back into the model to seek model parameter values which, jointly, provide an optimal fit to the spread of the experts’ evaluations. In the present case, probabilistic inversion seeks to find the model parameters which best represent, in an overall sense, the individual preference rankings of the experts for the likelihood of concern, and to quantify the collective scatter and randomness that are, inevitably, intrinsic to such ranking judgements (see applications in (Kraan and Cooke 2000; Fuller et al. 2017)). Numerical algorithms exist for solving this inversion, implemented in the program UNIBALANCE (Macutkiewicz and Cooke 2006); see Sect. 2.4 below).

The selected technique does not quantify the aforementioned likelihoods for each scenario and its associated uncertainty. Instead, the elicitation aims to identify the relative order of each scenario based on its likelihood of occurrence in a pre-defined period of time, and how likely it is to cause a given level of consequence and evaluate the degree of disagreement among the participants. Therefore, in this study, paired comparison has been selected as it has four main qualities:

1. Reproducibility, enabling peer review of the results;
2. Accountability, as all assessments are recorded and could be checked by a reviewer;
3. Neutrality, as the stakeholders are invited to complete the questionnaire individually and privately, after a reasonably comprehensive discussion of generic technical issues or ambiguities relating to the scenarios. As such, it reduces the potential for one or more participating stakeholders to unduly influence colleagues due to dominant personalities, or for ‘groupthink’ biasing;
4. Equity, as the participants’ opinions are not judged prior to their participation in the elicitation.

In addition to the above, the methodology that we propose ensures confidentiality of individual responses and anonymity of the participants (and their organizations) in the presentation of any specific findings. This encourages each participant to provide their own considered judgement without fear of criticism. It should also be mentioned that the adopted elicitation technique aims to perform a comparative assessment and for this reason, it lacks the empirical control when the aim is to quantify numerical uncertainty associated with a specific scenario. Thus, the participants are not assessed based on how well they are able to quantify uncertainties, per se [e.g., as in the Classical Model (Cooke 1991)] and given equal weights as a result.

**Fig. 1:**
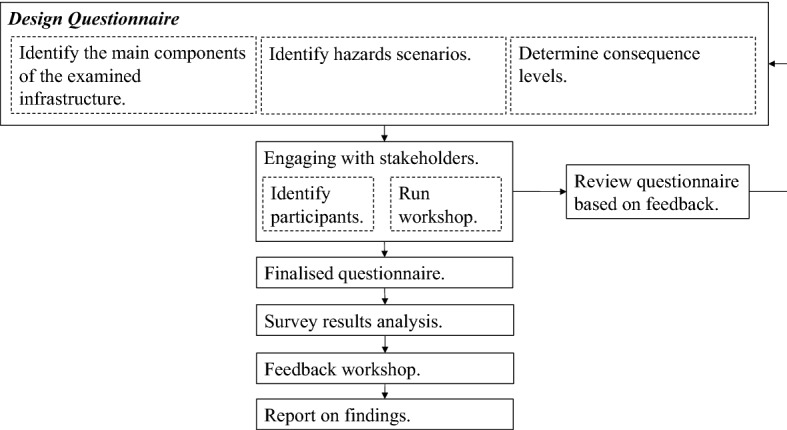
Framework of methodology to identify suitable hazard scenario for assessing the resilience of infrastructure

The main steps in the proposed methodology are illustrated in Fig. 1. The process starts with establishing a collaboration between the problem owners (who can be representatives of local government or operators of a group of infrastructures in a region) and the facilitator of the elicitation whose main responsibility is to adequately channel the former’s knowledge and needs and to minimize biases in the process. Key skills of the facilitator are their familiarity with the paired comparison technique, their ability to construct a survey avoiding biases, and their understanding of any applicable data protection regulations.

Prior to the elicitation, a number of stakeholders whose role is to help compile the list of scenarios, as well as rank them, must be identified. Suitable stakeholders could either be those who are familiar with the operation of a particular infrastructure, those who rely on its service, or operators of infrastructure in a region with experience in risk assessment. With the facilitator and stakeholders identified, the elicitation takes place. In what follows, a detailed description of each step is provided.

### Design of the elicitation questionnaire

The success of the elicitation depends on a well-designed questionnaire. To ensure its quality, a well-attended workshop with stakeholders is necessary to mitigate the potential of unknown, ignored or excluded scenarios and also to ensure that the questionnaire adopts terminology that is familiar to the stakeholders and is unambiguous in meaning for the specific assessment.

The elicitation questionnaire proposed has five main parts. The first part introduces the potential participants to the main objectives of the elicitation and describes why their insight is needed. In the next two parts, general professional information from the participants are requested, and a consent form can be found with information confirming that the study complies with any relevant ethics and data protection requirements. A clear assurance is also given that the participants’ responses will remain anonymous. The fourth part contains the main elicitation and includes a concise description of the problem, the main components of the examined infrastructure, the definition of the consequence levels and the list of the predefined scenarios. Moreover, the paired comparison technique is explained with clear and detailed instructions provided on the role of the participants. The fifth part invites the participants to provide their feedback on the elicitation and raise any concerns regarding the questionnaire. In what follows the material found in the fourth, (i.e., elicitation) part of the questionnaire is presented in detail.

#### Identification the types and components of assessed infrastructure

Infrastructure systems are complex, often spatially distributed, networks. As a first step, information is collected with the aim of gaining an in-depth understanding of the region being studied, including interaction between different infrastructure systems. To do this, a list of the major components of the various systems and/or networks and their main characteristics is constructed. The focus is on characteristics (e.g., location, age, design characteristics, construction materials) which are deemed important for the assessment of their vulnerability to different hazards. Finally, information regarding the functioning of the infrastructure is necessary to understand the importance of the services it provides at local, national and international level.

#### Identification of candidate hazard scenarios

**Fig. 2:**
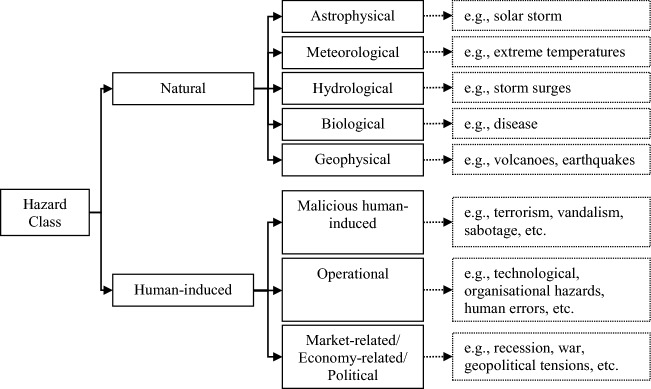
Classification of hazards for the needs of this study

A list of possible hazard scenarios that can affect the infrastructure is compiled. To assist the elicitation facilitator to compile these hazard scenarios, the methodology proposes the construction of scenarios for a given hazard class. In Fig. 2, hazard classes are depicted in broad agreement with precedents in the literature. The facilitator can select widely ranging scenarios from natural and human-induced hazard classes. The former class includes diverse hazards such as geological, astrophysical and hydrological. The latter class can include malicious human activities, operational hazards, market-related/economy-related and political hazards. This study focuses on the natural and operational hazards, which refer to accidents caused by human error as well as organizational or technological failures (e.g., non-compliance with safety procedures or failure of IT systems, etc.).

The selected hazard scenarios are mainly based on knowledge of past events that have affected the region, the specific infrastructure system or similar systems in other locations. The incorporation of scenarios adopted in the literature for the risk assessment of the examined type of infrastructure is also recommended. However, it is important not to concentrate only on past events that have caused large or disastrous consequences, but to include “near-miss” events that might be repeated at greater intensity in the future with catastrophic consequences, and to consider “counterfactual” events (Woo 2012, 2013) which, while having no exact historic precedent, under slightly different circumstances could represent plausible hazard scenarios.

#### Determination of consequence levels

Discrete levels of consequence also need to be determined to aid the comparison of different hazards and across different infrastructure systems. Table 1 presents an example of three consequence levels that can be defined for a generic infrastructure system. In general, a high frequency of minor incidents at a given infrastructure means that the operators are highly likely to be well prepared for them. Operators are less likely to be fully prepared to cope with less frequent and highly uncertain events, which can cause an emergency situation or even a disaster situation. For this reason, the application of the proposed methodology focuses on the consequence levels of emergency and disaster.

**Table 1:** Definition of consequence levels

Consequences level	Description
Disaster	A catastrophic consequence hazard event which causes major disruption to the infrastructure in the region and which has a severe impact to the cities it serves
Emergency	A medium consequence hazard event which causes severe disruption to the infrastructure in the region and a moderate impact to the cities it serves
Minor Incident	A localized low consequence hazard event which causes the partial disruption to the infrastructure in the region

#### The elicitation process

Having determined the list of hazard scenarios and the definition of the consequence levels, the elicitation process can take place, during which the participants are invited to provide their ‘contingent evaluations’ (sometimes called ‘stated preferences’) (Cooke and Misiewicz 2007) with three aims. The first aim is to rank the hazard scenarios of a given hazard class according to their likelihood to occur in a given timeframe. It should be noted that the timeframe is determined by the facilitator and it aims to constrain the problem and to assist the participants with their selection. For example, use of a very short timeframe, (e.g., one year), aims to identify hazard scenarios that the stakeholders consider as imminent to cause a given level of consequences. The second and third aim is to determine the relative order of each scenario according to their likelihood to cause emergency or disastrous consequences, respectively. Within the paired comparison method, for each hazard class, the scenarios are formatted into three preference matrix panels, laid out as depicted in Table 2. In this illustrative version of the preference matrix, the three different questions are summarized in the first cell. For each preference matrix, the participants are invited to compare every unique pairing of hazard scenarios according to the three aforementioned questions.

**Table 2:**
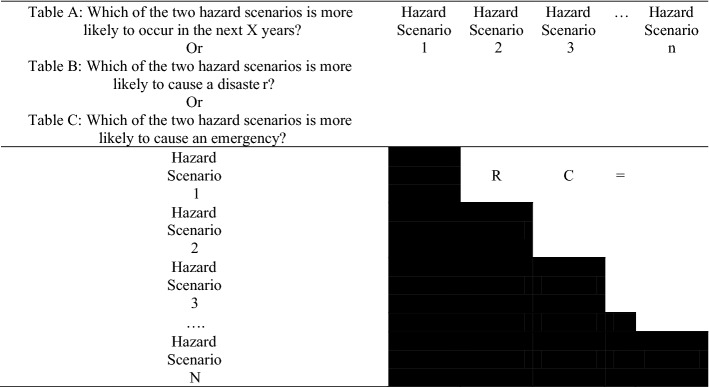
Generic table layout for paired comparison elicitation. There are three similar tables, *A*, *B* and *C*, one for each level of consequence and type of hazard

The procedure for completing each of the three preference matrices is as follows: The participating stakeholder is asked to consider the choice criteria in the top left cell and then to place in each of the empty boxes on the upper right half of the matrix their choice: an “*R*” indicating that the incident on the Row (Hazard Event 1) better meets the relevant criterion than the incident (Hazard Event 2) in the column; or a “*C*” if they think that the incident in the Column better meets the criterion. In a case where the expert’s opinion is that the two incidents are equally likely to meet the criterion then an equals sign (“=”) should be put in the box (as illustrated in Table 2). However, participants are discouraged from using this decision opt-out too frequently as it represents a potentially pathological absence of information for the probabilistic inversion algorithm.

### Engaging with the stakeholders

The role of the facilitator is to recruit suitable participants to elicit. One of the challenges faced by the facilitator is to engage with relevant stakeholders to participate in the study. As a first point of engagement with the stakeholders, a workshop is organized. To mitigate a potential low attendance, identified participants who are invited to the workshop are asked to invite further participants through their contact lists.

#### Identify participants

The number and background of the participants are also key contributors to the success of the elicitation. Guidance on expert elicitation procedures recommends that at least four experts be elicited, with eight being an optimum number of experts. The validity of the results depends not only on the number of participants but also on whether all different schools of thought in the subject area of interest are represented by the participants. A list of potential participants to be elicited is compiled. Suitable participants as discussed above could be stakeholders of the region or infrastructure in question or other suitable participants.

#### Pilot workshop

The pilot workshop is a first point of engagement with the stakeholders. It provides a means of tailoring and honing the initial questionnaire into a regionally collocated infrastructure resulting in a specific, useful, and more engaging tool for the participants and therefore reducing the risk of major disagreement with the results or critique of the questionnaire.

To meet the above aims, during the workshop information is provided on the objectives of the study, the need for the elicitation and the participants are familiarized with the paired comparison technique. The participants are presented with a list of pre-defined hazard scenarios and are invited to agree on a final list after discussing the usefulness of the pre-selected hazard scenarios, adding scenarios that they consider important and removing the scenarios that they collectively consider as irrelevant or unlikely to cause an emergency or disaster. From the practical application of this procedure, it is found that a maximum of 10–15 scenarios for each hazard class are adequate. The workshop is also used to remove possible ambiguities in the description of hazards, technical jargon or definitions of infrastructure components, consequences, etc., in the questionnaire. This process is important as terminology differs across infrastructure sectors, and across different cultures and countries.

#### Finalized questionnaire

The updated questionnaire is sent in an electronic form to the stakeholders as defined above. The participants are invited to complete the paired comparisons in their own time, without interacting with other participants. The questionnaire developed in this case study takes approximately 45 min to be completed. The completed questionnaires are returned by email or post to the facilitator.

### Analyses of findings

The qualitative data from the completed tables are analyzed using the software package ‘UNIBALANCE’ (Macutkiewicz and Cooke 2006), an analysis package that incorporates the probabilistic inversion technique to assess variances in the individual responses and in the collective responses of the group as a whole. This produces a best-estimate score for each hazard event rated by the participants as well as the standard deviation around this score which represents the level of disagreement within the expert group. The degree of consistency of both the individual participants and of the group of participants as a whole is also tested as part of the software analysis [see (David 1988) for more details on the tests performed and metrics adopted].

A chi-square test is performed by the software to test the null hypothesis that an individual expert appears to have responded randomly. It may be noted that filtering out an individual who gives random rankings is a weak form of empirical control. The test provides the *p* values, which below 0.05 indicate enough evidence in the responses to reject the hypothesis that the experts responded randomly. By contrast, *p* values well above the 0.05 threshold highlight a participant who completed the tables randomly. The confidence in the results is ensured by removing the response of those participants who are found to have completed the questionnaire incoherently as far as logical choice combinations are concerned.

With regard to the group of participants, their level of agreement as a group is examined in three different ways. Firstly, a chi-square test is conducted by the software to test the null hypothesis that the preferences expressed by the respondents, as a group, are random. For instance, from this test, a *p* value well above the 0.05 threshold might be taken to highlight the possibility that the set of responses fail to exhibit some degree of collective accord, within the group, regarding the ranking order of the hazard scenarios. This can happen if too many respondents provide illogical or irrational choices. Secondly, the ‘coefficient of agreement’ metric evaluates how closely the patterns of the individual stakeholders’ pairwise comparisons match one another. It is calculated by the software and ranges from 0 to 1: A value close to one indicates strong similarities that are present in the pairwise choice patterns the experts provide. Lastly, the ‘coefficient of concordance’ (which also ranges between 0 and 1) measures how closely the experts’ option rankings determined by the patterns of individual pairwise choices correspond to one another among the group of participants. For one particular set of pairwise comparisons, and on their own, these two metrics are only roughly indicative of the levels of agreement and concordance that exist. The metrics can be more informative, and potentially diagnostic, if the whole topic being addressed covers a number of different issues or factors for which expert pairwise choices are elicited; then, agreement and concordance scores can quantify the relative evidential strengths of each component of the pairwise elicitation, as contributions toward characterizing the make-up of the problem overall.

### Feedback workshop: results validation

Having analyzed the results, a feedback workshop with the participants is held to discuss the findings. The participants are invited to express their opinions on whether they agree or disagree with the findings and, if critical, provide justifications for their disagreements. The facilitator should be prepared for the extreme case of a majority of the participants expressing major disagreement with the outcomes. In this case, the questionnaire could be revised and sent to the participants for the process to be repeated.

### Report of findings

Reporting the findings is the final step of the proposed methodology. The report should contain the aggregated data and if desired dependent on the context can be structured to ensure the anonymity of the participants and their organizations.

## Case study application: the Øresund region

The case study presented in this paper is intended to demonstrate the application of this method to the infrastructure generally in the Øresund region, without specific reference or consideration to any infrastructure asset or sector. The general nature of this case study was explained to the participants at the outset. What follows is a brief description and a summary of the most significant infrastructure geographically co-located in the region, as well as of the region itself.

The Øresund region covers the region of Skåne on the Swedish side of the Øresund strait, the capital region of Denmark and Zealand region (islands Sjælland, Lolland, Falster, Møn and Bornholm) on the Danish side. With a total population of nearly 4 million people, the region is an excellent example of European cross-border collaboration and interdependency, building on the metropolitan area around Copenhagen and Southern Sweden, with the cities of Malmö, Lund and Helsingborg.

The most obvious infrastructure connecting the region is the Øresund fixed link, a four-lane motorway and rail (two tracks) bridge/tunnel across the Øresund between Copenhagen and Malmö, with associated motorway and railway connections. Denmark and Sweden’s transport infrastructures are interconnected with the Øresund road and rail link, which is used daily by over 10,000 commuters. The Øresund road and rail crossing comprises an 8 km long bridge spanning between a 4 km long artificial island in the middle of the sound and mainland Sweden and the 4 km long Drogden immersed tube tunnel between the artificial island and Copenhagen in Denmark. As well as linking two large communities by road and rail, allowing commuters to live on one side and work in the other, the crossing (along with other bridges in Denmark) is the primary road and rail link between Scandinavia and mainland Europe and has reduced the crossing time to a 15 min car or train journey. Train traffic via the Øresund Bridge is reliant on the electricity infrastructure of both Denmark and Sweden. At the time that this study was undertaken, the bridge also carried the main fiber optic data connection to Finland.

Other transport infrastructure in the region includes the Copenhagen airport, which is the sixth largest air transportation hub in Europe used daily by over 10,000 travelers, 4000 of whom travel for business; the ferry between Helsingør and Helsingborg; the navigational routes in the strait; several ports (e.g., Copenhagen, Malmö, Helsingør, Helsingborg, Landskrona); Copenhagen Metro and Malmö city tunnel; connected highways (e.g., E47, E20, E6); and the Øresund railway line.

In this case study, the most critical natural and operational hazard scenarios for the infrastructure in the Øresund region are identified using the proposed risk-prioritization framework. In what follows, a brief account of past natural events and operational accidents that have affected the Øresund region is presented, together with the results obtained by applying the proposed methodology developed. It should be mentioned that in the present case study, the stakeholders consulted were individuals or representatives of organizations who have a specific expertize and familiarity with the infrastructure of the Øresund region in general but did not represent any of the owners or operators of the infrastructure, as such the application presented is only exemplary. The authors were the facilitators. The goal of the study is to prioritize the hazardous scenarios for the region such that they could provide input to the regional risk assessment or to the risk assessment undertaken by the operators of the infrastructure in the region. This study also allows for an assessment of the level of agreement among the stakeholders, which captures the complexity of the problem as well as the extent of any lack of data or divergence of judgements. As in any hazard identification study or risk assessment, it is acknowledged that the selection of a sample of hazard scenarios can usually only represent a fraction of the range of scenarios that could occur. However, this does not reduce the value of the prioritized list of hazardous scenarios that can be obtained by applying the methodology.

A questionnaire containing three tables with the generic form of Table 2, for the pre-selected natural and operational hazards (see Tables 3 and 4) was completed by eight participants altogether, with experience in assessing natural and operational hazard risks in the Øresund region. Half of these participants had experience of the Swedish part of the Øresund region and the other half had experience of the Danish part of the region. The participants provided their pairwise preferences regarding the likelihood of natural or operational hazard scenarios occurring in the next 5 years in the region, as well as the likelihood that they would cause a disaster or emergency. Participants and their affiliations are kept anonymous.

**Table 3:** Natural hazards scenarios used for the structured exert elicitation with the corresponding mean rank score and standard deviation

No.	Natural hazard Scenario	Likelihood of								
		Occurrence			Disaster			Emergency		
		Option	Mean	SD	Option	Mean	SD	Option	Mean	SD
1	Earthquake	√	0.15	0.113	√	0.22	0.225	√	0.15	0.114
2	Storm surge	√	0.41	0.242	√	0.58	0.271	√	0.46	0.309
3	Extremely high winds	√	0.66	0.195	√	0.77	0.164	√	0.79	0.145
4	Extreme temperatures (high)	√	0.42	0.268	√	0.41	0.214	√	0.42	0.211
5	Extreme temperature (low)	√	0.40	0.158	√	0.45	0.198	√	0.46	0.217
6	Lightning:	√	0.74	0.162						
7	At the rail network									
	On a ship/ferry									
	At the airport									
	Causing a major power outage				*√*	0.68	0.279	√	0.71	0.220
8	Snow Storm	√	0.48	0.207	√	0.68	0.196	√	0.70	0.176
9	Solar Storm	√	0.12	0.088	√	0.44	0.312	√	0.37	0.288

Statistics										
1	Coefficient of agreement	0.411		0.147		0.279				
2	Coefficient of concordance	0.673		0.331		0.505				
3	Random preferences *p* value (group-ranking)	0.000	Reject random rank scores hypothesis	0.000	Reject random rank scores hypothesis	0.000	Reject random rank scores hypothesis

**Table 4:** Operational hazards scenarios used for the structured exert elicitation with the corresponding mean rank score and standard deviation

No.	Operational hazards scenario	Likelihood of					
		Occurrence		Disaster		Emergency	
		Mean	SD	Mean	SD	Mean	SD
1	Pandemic (at least 25% of staff off sick)	0.22	0.156	0.66	0.265	0.62	0.289
2	Cargo ship/ferry accident in the strait (e.g., foundering, grounding, collision, fire/explosion, loss of stability)	0.56	0.317	0.32	0.258	0.27	0.198
3	Aircraft collision with one of the bridge pylon towers	0.18	0.174	0.50	0.288	0.54	0.272
4	Hazardous goods accident on the road deck of the bridge/in the tunnel	0.54	0.245	0.61	0.286	0.56	0.246
5	Serious road accident on the bridge/in the tunnel (not including hazardous goods)	0.69	0.238	0.51	0.280	0.52	0.243
6	Hazardous goods accident on the rail deck of the bridge/in the rail tunnel	0.47	0.249	0.58	0.266	0.54	0.274
7	Serious rail accident on the bridge/in the tunnel (not including hazardous goods)	0.59	0.247	0.55	0.252	0.52	0.287
8	Major off-site power outage for 8 h or more	0.47	0.275	0.45	0.290	0.47	0.278
9	Airside accident at airport (e.g., aircraft collision, accident at fuel farm)	0.42	0.240	0.57	0.255	0.66	0.298
10	Landside accident at airport (e.g., fire in the terminal building)	0.65	0.284	0.32	0.228	0.30	0.220
Total No. of scenarios	10	10	10				

Statistics							
1	Coefficient of agreement	0.12		0.00		0.00	
2	Coefficient of concordance	0.37		0.16		0.17	
3	Random preferences *p* value	~ 0.00	Reject random rank scores hypothesis	1.00	Cannot reject random rank scores hypothesis	1.00	Cannot reject random rank scores hypothesis

In the following, a detailed discussion of the results depicted in Tables 3 and 4 is presented, and the critical scenarios that may be used to assess the disaster and emergency resilience of the Øresund region to natural and operational hazards are identified.

### Natural hazard scenarios

No major disaster has hit the Øresund region in the recent past. Nonetheless, several natural hazard classes have caused various levels of disruption to the operations of the region’s infrastructure.

Both Copenhagen and Malmö are low-lying coastal cities. Their location increases their vulnerability to flooding. In 2010 and 2014, heavy torrential rains caused flooding and closures of roads in Malmö and other parts of the Skåne region (The local 2010, 2014). In 2011, flooding also damaged homes and caused disruption in parts of Copenhagen (BBC 2011). In August 2014, heavy rains caused flooding and train delays in Copenhagen (Radio Sweden 2014a, b, c). Storms and strong winds are also found to represent prominent hazards in the region. Between 2014 and 2016, three storms (i.e., storm Urd, Egon and Alexander) forced the closure of the Øresund bridge (Radio Sweden 2014a, b, c; Radio Sweden 2015; The local 2016). In 2013–2014, two strong storms (i.e., storm Simone and Sven) struck the Øresund region causing extensive damage to the infrastructure of the two countries and billions of kronor in insurance claims (Local 2013; Radio Sweden 2014a, b, c). The storms also caused problems in ferry transport between Denmark and Sweden and had a negative impact on the fishing industry (Local 2013; Radio Sweden 2014a, b, c). In 2011, icy conditions forced the temporary closure of the Øresund bridge in order to prevent possible damage to passing cars due to ice from the cables falling on the deck of the bridge (Radio Sweden 2011). With regards to the geological hazards, the Skåne region is known for its extremely low seismic activity (Voss et al. 2009). There have been 14 earthquakes since 1375 with only three small earthquakes (each less than magnitude 2.8) detected between 1970 and 2008. A ‘moderately strong’ event with magnitude 4.2–4.3 (Arkert 16/12/2008) occurred at 06.20 am CET on 16 December in 2008, which affected the southern part of Sweden and eastern parts of Denmark. The epicenter was 60 km east of Malmö. Finally, a solar storm has hit Denmark although without causing damage.

In Table 3, the nine natural hazard scenarios used in the elicitation are depicted. Based on the results of the research into past incidents, these scenarios include storm surge, snowstorm, extremely high winds, extreme temperatures (both low and high), an earthquake and solar storm. The scenario of lightning is also included as it can cause damage to the transport infrastructure in the region either by directly striking the rail network, the airport, a ship/ferry or by causing a major power outage in the region. The railway infrastructure on the Øresund link is based on the electricity network of both countries. Therefore, a power outage will cause traffic disruption in one or both countries. It should also be noted that the constructed hazards scenarios are generic and, with the exception of lightning, they are considered to affect most of the Øresund region around the crossing.

The natural hazard scenarios listed in Table 3 are then ranked using the pairwise preferences of the eight participants. It is highlighted that despite multiple lightning strike scenarios, the discussion of the results is limited to lightning that causes power outage, which is found to have the highest mean rank score of all the alternatives regarding the likelihood of these scenarios to cause disaster or emergency.

Prior to a deeper analysis of the results, it is important to test the consistency of the responses for individual as well as for the group of participants. A chi-square test is used to investigate whether each individual participant stakeholder stated their preferences randomly. In Table 3, the *p* values produced by a statistical test are presented for the eight participants for the three tables. The values are very close to zero and lie below the 0.05 threshold, indicating that the participants are not providing random or incoherent pairwise preferences.

With regard to the consistency of the group of participants, it is observed in Table 3 that the *p* values also lie below the 0.05 thresholds indicating that the group-rankings of all three likelihoods (i.e., of Occurrence, Disaster and Emergency) are not random. With respect to the coefficients of agreement and concordance, these values (see Table 3) are higher for the likelihood of occurrence than their counterparts for the likelihood of disaster or emergency. This indicates there is a higher degree of agreement among the participants about the ranking of the hazard scenarios according to their likelihood of occurrence, and less agreement on their ranking according to their likelihood to cause disaster or emergency to the Øresund region. This may be rooted in differences in knowledge about specific infrastructure arising from disciplinary or experiential differences of the participants. However, bearing in mind that the context of the study is the region as a whole, this can be argued as a positive feature since it reflects the potential impacts across the entire region.

**Fig. 3:**
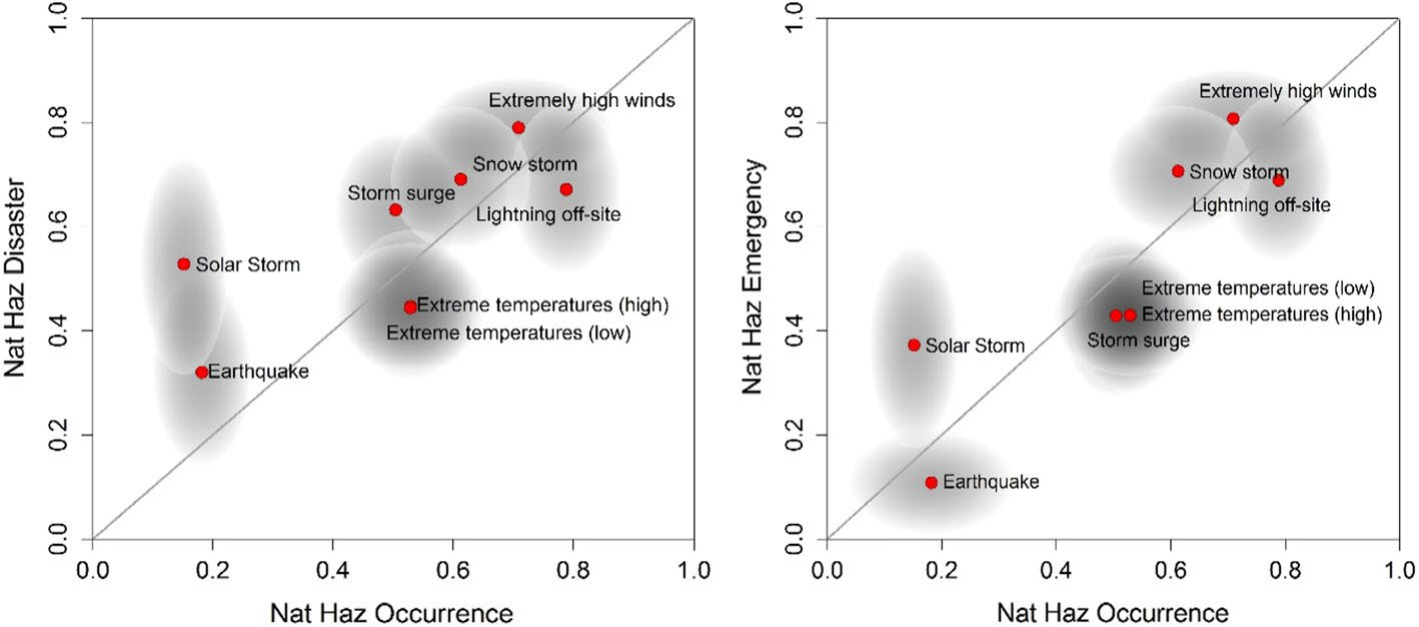
Plots of the rank scores of the natural hazards according to their likelihood to occur in the next 5 years against their likelihood to cause Disaster (left) and Emergency (right) in the Øresund region

Figure 3 depicts the plot of the mean rank score of the natural hazard scenario regarding their likelihood of occurrence against their ranking regarding their likelihood to cause Disaster or to create an Emergency. If the event occurrence rankings and the consequence rankings are identical, then the scenario points (red dots) plot on the diagonal line. If, however, there are differences in rank order, in either scenario occurrence, consequence or both, then those points will plot off-diagonal and are easily identified. Note also that, on Fig. 3, the rank order scores are normalized to span from 0 to 1, so any close clustering together of different scenario rankings is clearly manifested. The level of agreement among the group in the relative position of each scenario is also presented in terms of an ellipse around each mean score, which expresses the 95% confidence area estimated from the standard deviation of the rank scores. A smaller scenario ellipse diameter in one axis direction implies a higher degree of agreement among the participants for the relative position of this scenario on that axis compared to the position in the other dimension.

In Fig. 3, most scenarios appear to map on or close to the diagonal. The most obvious exception is Solar Storm, which has a low probability of occurrence but higher rank scores for Disaster and Emergency. The apparently binary consequences of the Earthquake scenario are also noteworthy: a much higher rank score for Disaster compared to Emergency. One interpretation of this finding could be that a high magnitude earthquake could have the potential to cause significant even disastrous damage, whereas at lower magnitudes, effects would be almost inconsequential. In other words, there is a marked cliff edge for earthquakes beyond which they escalate from marginal likelihood of Emergency to a more significant likelihood of causing a Disaster.

Figure 3 also depicts a notable degree of disagreement in the relative order of each scenario, which is not surprising given the complexity of the task and the large uncertainties. This indicates that the participants are in overall agreement that the ranking of the scenarios according to their likelihood of causing Disaster or Emergency is similar to their ranking according to their likelihood to occur in the next 5 years. This highlights that the participants are more interested in the frequent, (in relative terms), natural hazards than the less frequent hazards. Overall, the participants highlighted their concern regarding the meteorological hazards, which rank high in both their likelihood and their impact in the Øresund region.

In particular, the scenarios of extremely high winds, snowstorm and off-site lightning causing a power outage are the three scenarios which are considered both more likely to occur in the next 5 years and more likely to cause Disaster. It should be noted that storm surge also ranked very high in the likelihood to cause Disaster, although it ranked 7th in its likelihood to occur in the next 5 years. A notable outcome of the elicitation is the scenario of the solar storm which appears to be more likely to cause Disaster than to cause Emergency. For this scenario, the large radius of the ellipse in the y-direction regarding its likelihood to cause Disaster can also be noted. This means that the participants are not in agreement regarding its ranking order. Despite this, the solar storm scenario can also be flagged as potentially critical given that its rank score could be as high as the snowstorm or the off-site lightning.

In contrast to the complex picture of the disaster-level, the plot of rank scores of likelihoods of Emergency against the scenario likelihoods to occur in the next 5 years shows less significant off-diagonal deviations. In particular, the three meteorological scenarios, namely extreme high winds, snowstorm and lightning, are identified as the hazards most likely to occur and cause an Emergency in the region. This said, the degree of dispersion in the exact ranking order of each of the three scenarios is sizeable and identifying a single dominant hazard scenario on this basis is, therefore, difficult.

### Critical operational hazard scenarios

The transport infrastructure in the Øresund region could also be disrupted by operational hazard scenarios. News reports over the recent years showed that since its opening 17 years ago, the Øresund link had to be closed on multiple occasions due to road accidents. Notably, on the 15th February 2017, the link was closed after at least 10 cars were involved in an accident which injured 14 people (The local 2017). Nonetheless, the structural integrity of the link is protected by a careful design (Lykke et al. 1998; Hauge and Petersen 1999), which includes events such as fire in the tunnel or ship collision to the bridge piers, and has yet to be challenged. Broader research in major incidents in the Øresund region identified fire as a prominent hazard. Most notably, in 2013, a fire destroyed a recycling station in Malmö’s Norra Hamnen. The next year, a fire in a sugar factory in Malmö, led several thousand tons of sugar to melt and to spill outside the factory before being put under control by the Swedish firefighters three days later.

In Table 4, the 10 operational hazard scenarios used in the elicitation are presented. Half of these scenarios are expected to affect directly the Øresund link and they concern accidents on the bridge or the tunnel. In order to investigate how the stakeholders rank the operational risk of different infrastructure assets in the region, three of the considered hazard scenarios directly affect the airport and the ship/ferries in the Øresund strait. The remaining two scenarios are expected to affect the region as a whole and include a power-outage and a pandemic. It is noted that this elicitation was conducted in 2016 before the Coronavirus (COVID-19) pandemic affected the globe in 2020.

The 10 hazard scenarios are ranked using the UNIBALANCE (Macutkiewicz and Cooke 2006) probabilistic inversion results according to their likelihood of Occurrence in the next 5 years and their likelihood to cause Disaster or Emergency in the Øresund region.

The consistency of the responses of each individual participant and the degree of agreement within the group of participants are assessed first. Individually, the eight individual stakeholders appear to have provided consistent and non-random pairwise preferences for all three likelihoods.

Unlike the relatively clear picture in the natural hazards scenarios, the ranking of the operational scenarios is associated with high degree of disagreement among the participants. This is reflected in group ranking metrics. In particular, the approximately zero *p* value (see Table 4) suggests that there is enough evidence to reject the random rank scores hypothesis which means the group-wide ranking order is coherent only for the likelihood of occurrence. By contrast, the very high *p* values indicate that the group-wide ranking order is incoherent for the likelihoods of Disaster and Emergency. In line with this, the low values of the coefficients of agreement and concordance for the likelihood of Disaster and Emergency also suggest a large degree of dissimilarity among the participants’ patterns of pairwise preferences, as well as in the collective ranking order of the different scenarios.

**Fig. 4:**
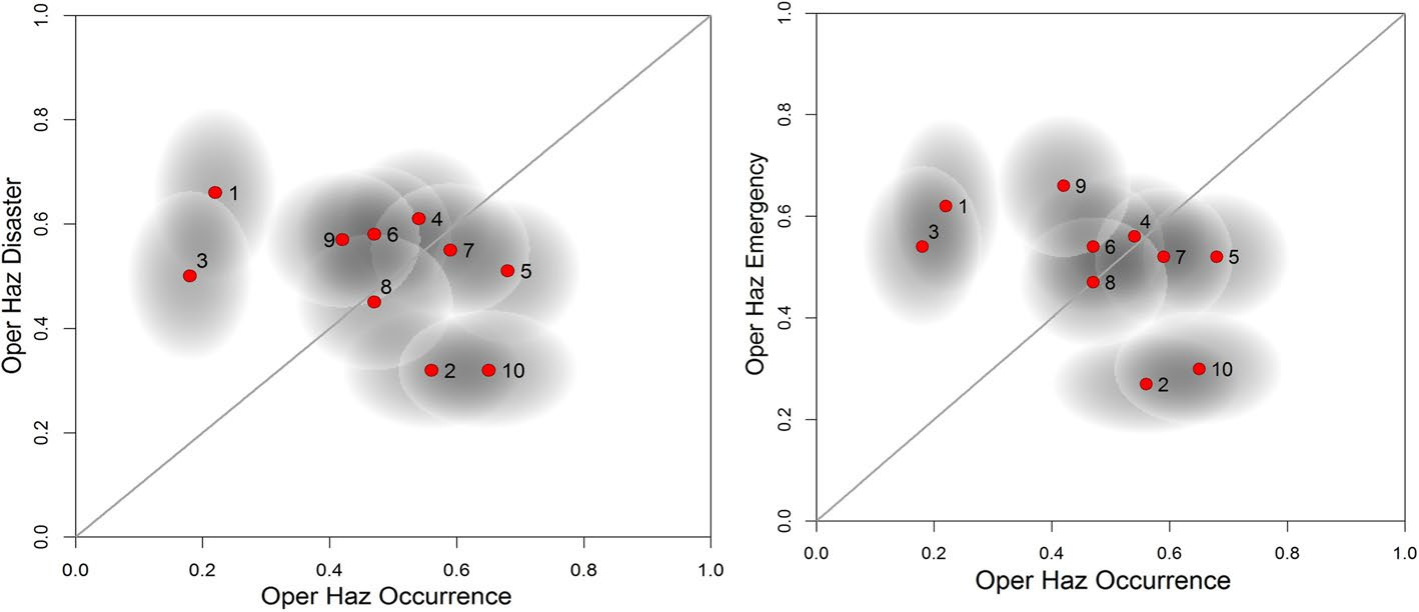
Plots of the rank scores of the selected operational hazards according to their likelihood to occur in the next 5 years against their likelihood to cause disaster (left) and emergency (right) in the Øresund region (see Table 6 for number keys)

The high degree of disagreement among the participants regarding the rank order of the scenarios is clearly highlighted by the large and overlapping ellipses around each rank score for the majority of scenarios in both direction in Fig. 4. The clustering of nearly all operational hazard scenario occurrences around rank score 0.5 reflects the fact that there is practically no agreement among the participants for differentiating likelihoods of occurrence or of causing emergency or disaster for most scenarios in the next 5 years. This suggests a significant lack of knowledge among the participants about the relative likelihoods of these events, which is, perhaps, not surprising given the lack of data regarding the risk of these type of events. If these clustered scenarios prevented the identification of clear high priority scenario’s, then a feedback workshop with the participants would explore possible explanations for the high uncertainty and through this identify whether reframing the problem could reduce the high levels of disagreement. This approach, however, would not guarantee the reduction in the uncertainties which could be inherent to the problem.

In our results, there are two outliers with significantly lower probabilities of Occurrence in both plots: 1—Pandemic, and 3—Aircraft collision with tower pylons; both markers rank quite high on the Disaster and Emergency scales, but the elongated ellipses indicate significant uncertainties associated with the rank scores of both. Similarly, the airside accident scores the highest mean score (in Table 4) but is also associated with high uncertainty. By contrast, the stakeholders appear to be in relative agreement that the accidents of a cargo ship or ferry at the straits or a landside accident at the airport are the least critical for the next 5 years.

The high degree of disagreement regarding the ranking order of the scenarios highlights the difficulty in prioritizing operational hazard scenarios. This is perhaps unsurprising given the diverse types of infrastructure considered and more discussion is required to address the reasons behind this disagreement. Despite this difficulty, it can be argued that in assessing the risk of the Oresund region, the scenarios of pandemic and aircraft collision are highlighted as worthy of attention for the Disaster case and for the Emergency the airside accident should be added to the aforementioned scenarios. Interestingly, the recent coronavirus pandemic which broke 4 years after the elicitation vindicated the stakeholders for flagging the pandemic scenario as an event least likely to occur than its alternatives but likely to have a severe impact in the region short term.

## Conclusions

A methodology is proposed for identifying the most critical natural or operational hazard scenarios in respect of disaster risk or emergency risk for different facilities, systems and assets comprising an infrastructure system. The novelty of the proposed methodology is the use of a structured expert elicitation procedure to obtain quantitative relative risk rankings. The proposed procedure, termed paired comparison, allows for the quantification of the degree of agreement or dissimilarity among the stakeholders in judging the rank order of the scenarios and consequences. The critical scenarios identified and the process of elicitation, which comprises stakeholder workshops, is found to foster discussion among relevant but diverse stakeholders toward improved cross-sectoral emergency and disaster planning. The methodology allows characterizing the stakeholders perception which enables prioritizing scenarios as well as identifying need for further knowledge development. The latter is particularly relevant when scenarios cluster and have large uncertainty. Overall, this characterization is in itself a key element to foster risk communication and to take stakeholder’s views into account in structuring risk management and resilience plans.

The proposed methodology was applied to identify critical hazard scenarios for the Øresund region. The methodology was successful in engaging with stakeholders and identifying critical scenarios taking into account their degree of disagreement. Overall, the participants found it easier to reach a consensus in the rank order of the natural hazard scenarios and struggled to reach a consensus in the ranking of the operational ones. With regard to the natural hazard scenarios, the participants highlighted as critical mostly meteorological scenarios. In particular, the relatively frequent events such as extremely high winds, snowstorm and off-site lightning were identified as critical for both the Disaster and Emergency risks. With regard to Disaster risk, the participants also identified storm surge as critical and the solar storm scenario was also flagged as potentially critical. With regard to the operational hazard scenarios, despite significant disagreements among participants, the scenarios of pandemic and aircraft collision were highlighted as potentially critical for both levels of consequences. In particular for the case of Emergency, the airside accident was also flagged as potentially critical by the participants.

The disagreement among participants noted in the application is in many respects not surprising if there were unambiguous dominant hazard scenarios or individual risks, then an expert elicitation would not be needed. What the present methodology offers, for the transnational Øresund region, is a set of objective comparative rankings of natural and operational hazard scenarios and associated likelihoods of emergencies or disasters in the next five years, against which response planning and other priorities can be set, basic similarities in effect scale and impact magnitudes notwithstanding. It is believed that this process of paired evaluation, stakeholder engagement, workshops, discussion and feedback provide a means for resilience to be discussed and to raise awareness in stakeholders of potential risks.

## Data Availability

The anonymized responses to the questionnaire are available on request.

## Code availability

The freely available UNIBALANCE has been used for the analysis of the anonymized data. The code is available on request.
